# Intra-discal injection of autologous, hypoxic cultured bone marrow-derived mesenchymal stem cells in five patients with chronic lower back pain: a long-term safety and feasibility study

**DOI:** 10.1186/s12967-016-1015-5

**Published:** 2016-09-01

**Authors:** Christian Elabd, Christopher J. Centeno, John R. Schultz, Gregory Lutz, Thomas Ichim, Francisco J. Silva

**Affiliations:** 1 BioRestorative Therapies, Inc., 40 Marcus Drive, Suite One, Melville, NY 11747 USA; 2 The Centeno-Schultz Clinic, 403 Summit Boulevard, Unit 201, Broomfield, CO 80021-8253 USA; 3 Department of Physiatry, Hospital for Special Surgery, 429 E 75th Street, 3rd Floor, New York, NY 10021 USA; 4 Institute for Molecular Medicine, Huntington Beach, CA 92649 USA

**Keywords:** Regenerative medicine, Cell therapy, Degenerative disc disease, Autologous, Hypoxic, Mesenchymal stem cells, Bone marrow, Intra-discal injection, Safety, Feasibility

## Abstract

**Background:**

Chronic low back pain due to disc degeneration represents a major social and economic burden worldwide. The current standard of care is limited to symptomatic relief and no current approved therapy promotes disc regeneration. Bone marrow-derived mesenchymal stem cells (MSCs) are easily accessible and well characterized. These MSCs are multipotent and exhibit great tissue regenerative potential including bone, cartilage, and fibrous tissue regeneration. The use of this cell-based biologic for treating protruding disc herniation and/or intervertebral disc degeneration is a promising therapeutic strategy, due to their known regenerative, immuno-modulatory and anti-inflammatory properties.

**Methods:**

Five patients diagnosed with degenerative disc disease received an intra-discal injection of autologous, hypoxic cultured, bone marrow-derived mesenchymal stem cells (15.1–51.6 million cells) as part of a previous study. These patients were re-consented to participate in this study in order to assess long-term safety and feasibility of intra-discal injection of autologous, hypoxic cultured, bone marrow-derived mesenchymal stem cells 4–6 years post mesenchymal stem cell infusion. The follow-up study consisted of a physical examination, a low back MRI, and a quality of life questionnaire.

**Results:**

Patients’ lower back MRI showed absence of neoplasms or abnormalities surrounding the treated region. Based on the physical examination and the quality of life questionnaire, no adverse events were reported due to the procedure or to the stem cell treatment 4–6 years post autologous, hypoxic cultured mesenchymal stem cell infusion. All patients self-reported overall improvement, as well as improvement in strength, post stem cell treatment, and four out of five patients reported improvement in mobility.

**Conclusion:**

This early human clinical data suggests the safety and feasibility of the clinical use of hypoxic cultured bone marrow-derived mesenchymal stem cells for the treatment of lower back pain due to degenerative disc disorders and support further studies utilizing hypoxic cultured bone marrow-derived stem cells. The overall improvements reported are encouraging, but a larger double-blind, controlled, randomized clinical study with significant number of patients and implementation of validated endpoint measurements are next steps in order to demonstrate efficacy of this cell-based biologic.

## Background

Chronic low back pain affects approximately 632 million people worldwide and has an enormous social impact on patients and a significant economic impact on healthcare budgets [1]. In the United States alone, the annual cost is estimated to be as high as 500 billion dollars [2, 3]. The exact causes of disc degeneration are complex and involve genetic risks, smoking, obesity, aging and mechanical trauma [4]. The intervertebral disc is an immune-privileged avascular organ composed of fibrocartilaginous tissues that connect the vertebral bodies and provide stability, while allowing motion between the vertebrae [5, 6]. The centrally located nucleus pulposus is a hypoxic, hydrophilic proteoglycan-rich gelatinous core, surrounded by a lamellated collagenous ring, the annulus fibrosus and bony end-plates that separate the discs from the vertebrae [4, 7]. In a normal disc, there is a balance between the anabolic and catabolic capacity of the disc cells, but excessive stress can alter this balance and compromise extracellular matrix degradation and synthesis thereby initiating a degenerative cascade [8]. A loss in the cellular component function induces alterations in both the cartilaginous and proteoglycan matrix of the disc [4, 9, 10]. Subsequently, alterations of the proteoglycan component ultimately leads to dehydration of the nucleus pulposus, which impacts the ability of the disc to effectively distribute and recover from mechanical loading. The degenerated disc may eventually weaken to develop a protrusion towards the spine that puts pressure on the nerve roots or parts of the spinal canal, causing back pain and possibly weakness and numbness.

The standard of care for treating disc degeneration involves conservative non-surgical approaches or surgical interventions that target symptomatic relief and musculoskeletal stabilization. Currently, there is no clinical therapy targeting the reversal of disc degeneration or that addresses intervertebral disc cell homeostasis [11, 12]. The regenerative medicine field using cell-based therapies and biologics has advanced significantly over the past decade with numerous clinical trials aiming at providing treatments for neurodegenerative, orthopedic and cardiovascular disorders [11, 13–15]. Mesenchymal stem cells (MSCs) are stem cells that have extensive proliferative capacity and multi-lineage potential. They arise from progenitor cell reservoirs and have been isolated from various tissues, including bone marrow [16], muscle [17], and adipose tissue [18, 19]. MSCs isolated from various tissues share common characteristics, including the ability to: (1) form fibroblastic like colonies; (2) proliferate extensively; (3) be directionally differentiated into adipocytes, osteoblasts and chondrocytes; and (4) express several common cell surface markers. In addition, they possess immunomodulatory properties [20, 21] and have the ability to secrete a variety of cell mediators that aid in cellular repair and homeostasis.

The ideal treatment for degenerative disc disease (DDD) would be to conserve the physiologic function of intervertebral discs and reverse the degenerative cascade. The potential for stem cell-based therapy to treat disc degeneration is to repopulate the disc with viable cells capable of producing extracellular matrix, restoring damaged tissue and enhance tissue regeneration by the secretion of growth factors to modulate the inflammatory response.

In this short report, we studied the long-term safety and feasibility (n = 5) of intra-discal injection of autologous, hypoxic cultured bone marrow-derived mesenchymal stem cells for the treatment of chronic lumbar radiculopathy.

### Rational for hypoxic cell therapy

The intervertebral disc is the largest avascular tissue in the human body, with oxygen gradients ranging between 1 and 5 % [22]. Nutrients such as glucose and oxygen diffuse from the capillaries across the cartilaginous endplate and through the dense disc matrix to the cells. During disc degeneration, structural changes and vascular depletion reduce oxygen diffusion thus oxygen concentration lowers, resulting in further extracellular matrix degradation, ultimately causing a vicious cycle of disc degeneration.

Several studies suggested that hypoxic pre-conditioning has beneficial biological effects that can impact the therapeutic activity of MSCs in the disc. Hypoxic culture has been demonstrated to upregulate chondrocyte specific genes such as aggrecan, collagen type II and Sox-9 [23], upregulate the expression of hypoxia inducible factor-1α (HIF-1α), increasing the anti-apoptotic capabilities of MSCs [24–26] and activating focal adhesion kinase (FAK), which increases migration and homing [27]. In addition, MSCs cultured from older patients under hypoxia demonstrated enhanced self-renewal and proliferation capacity compared to cultures under normoxic conditions [28]. Hypoxic culturing of MSCs produces many desirable biological effects that may impact the therapeutic activity of the MSCs post-transplant into the limited nutrient, low oxygen tension microenvironment of the degenerative disc.

## Methods

### Study overview

The five patients described in this study where diagnosed with lumbar degenerative disc disease associated with protruding discs. Thirty-eight subjects were screened, seven patients were enrolled in the study and 5 patients completed the study. These patients, in procedures performed in the United States, received autologous, hypoxic cultured, bone marrow-derived mesenchymal stem cells under IRB protocol (IRB protocol# 00002637) between 2009 and 2010. All of these patients had failed lower back pain standard of care and pain management including oral medication, physical therapy, fluoroscopically-guided epidural steroid injections, and activity modification. Four to six years post-cell transplant (Table 1), these patients were re-consented to participate in this study under IRB protocol (IRB protocol #1140608) in order to evaluate long-term safety and feasibility of intra-discal injection of autologous, hypoxic cultured bone marrow-derived mesenchymal stem cells. This study included a follow-up examination consisting of a physical examination, the completion of a quality of life questionnaire, and a spine MRI performed and interpreted by an independent radiology group (Table 2).

**Table 1:** Summary of treated patients

Patient	Route	Disc injected	Total MSCs injected (millions)	Follow up (years)	Adverse events
1	Intra-discal	L5–S1	27.7	5	None
2	Intra-discal	L5–S1	51.6	6	None
3	Intra-discal	L5–S1	23.7	4	None
4	Intra-discal	L5–S1	15.1	5	None
5	Intra-discal	L4–L5	35.9	5	None

**Table 2:** Safety parameters

Patient	Physical exam	MRI	QoL^a^
1	X	X	X
2	X	X	X
3	X	X	X
4	X	X	X
5	X	X	X

^a^ *QoL* quality of life questionnaire

### Patients' inclusion and exclusion criteria

The five patients met ALL of the following criteria prior to receiving the injection of hypoxic-cultured MSCs:

1. Voluntary signature of the IRB approved informed consent.
2. Skeletally mature male or females between the ages of 18 and 65.
3. Pain, spasm, or functional disability in the low back and diagnosed as having painful DDD and having failed conservative treatments (e.g. NSAIDs, physician initiated physical therapy) for at least 3 months, but no longer than 5 years.
4. Significant functional disability related to pain, weakness, restricted range-of-motion or other back symptoms.
5. Either a painful annular fissure that is c/w physical exam and low pressure positive discography (painful P2 concordant response at less than 20 mmHG over opening pressure) or with or without Modic endplate changes.
6. If lumbar radiculopathy is present, it is due to a chemical radiculitis secondary to an annular fissure or small protrusion.
7. Is independent, ambulatory, and can comply with all post-operative evaluation and visits.

Candidates will be excluded if they meet ANY of the following criteria:

1. Symptomatic severe central canal or foraminal stenosis.
2. Previous history of lumbar spine surgery.
3. Prior epidural steroid injection within 12 weeks of the intra-discal procedure.
4. Lumbar radiculopathy caused by mechanical compression.
5. Spondylolisthesis/spondylolysis.
6. Inflammatory or auto-immune based pathology (e.g., rheumatoid arthritis, systemic lupus erythematosus, psoriatic arthritis, polymyalgia, polymyositis, and gout pseudogout).
7. Quinolone or statin induced myopathy/tendinopathy.
8. Severe neurogenic inflammation of the cutaneous nerves.
9. Condition that represents a workers compensation case or pending litigation.
10. Pregnancy
11. Bleeding disorders
12. Currently taking anticoagulant or immunosuppressive medication.
13. Allergy or intolerance to study medication.
14. Use of chronic opioids.
15. Documented history of substance abuse within 6 months of treatment.
16. Any other condition, which in the opinion of the investigator, would preclude the patient from enrollment.

### Isolation and hypoxic culture of autologous, bone marrow-derived mesenchymal stem cells

Bone marrow was harvested from each patient, as previously described [29]. Briefly, patients were placed in the prone position and 30 mL of bone marrow was harvested from each side of the posterior superior iliac spine area. The bone marrow aspirate was then centrifuged at 200×*g* for 6 min. The mononuclear cell layer was isolated and washed by centrifugation using phosphate buffered saline (PBS). Nucleated cells were plated into tissue culture flasks at a density of 1 × 10^6^ cells/cm^2^ and cultured in Dulbecco’s modified eagle medium (DMEM) with 10 % platelet lysate, 5 μg/mL doxycycline, and 2 IU/mL heparin in a 37 °C/5 % CO_2_/5 % O_2_ incubator. Mesenchymal stem cells were expanded under hypoxic conditions and prepared for injection.

### Injection of hypoxic cultured autologous, bone marrow derived mesenchymal stem cells

On the day of injection, hypoxic expanded mesenchymal stem cells were harvested, washed and re-suspended in autologous platelet lysate at a final volume ranging between 0.25 and 1 mL. Under c-arm fluoroscopic guidance, a 22 gauge spinal needle was placed over the inferior articular process of the targeted spinal level to enter the annulus fibrosus of the intervertebral disc. The needle tip was then positioned on biplanar fluoroscopy in the middle of the nucleus pulposus (NP). A small amount of contrast was injected into the NP to demonstrate a nucleogram and filling of the annular fissure, followed by the intra-discal injection of the mesenchymal stem cells at the indicated cell quantity (Table 1).

## Results

**Fig. 1:**
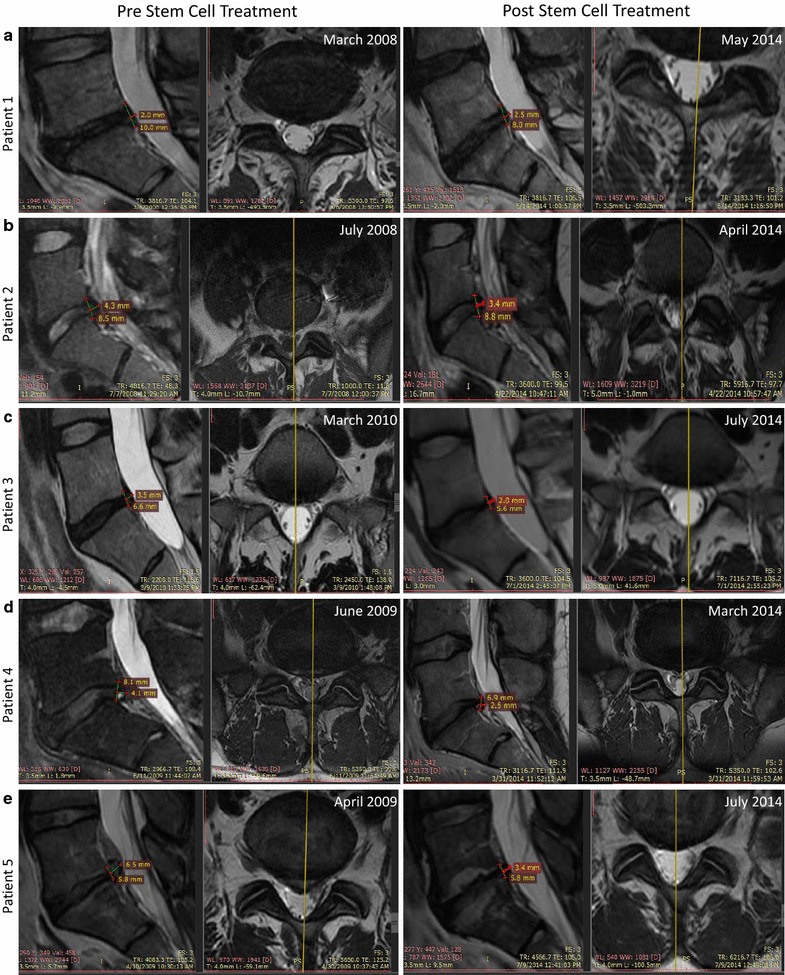
Patients MRI pre- and post- autologous, hypoxic cultured MSCs intra-discal injection. MRI pre- (*left panels*) and post- (*right panels*) stem cell treatment are shown for patient 1 (**a**), patient 2 (**b**), patient 3 (**c**), patient 4 (**d**), and patient 5 (**e**). Measurements of the posterior disc height and protrusion size at the L5–S1 level are indicated on each image as well as the date at which the MRI imaging was performed

**Fig. 2:**
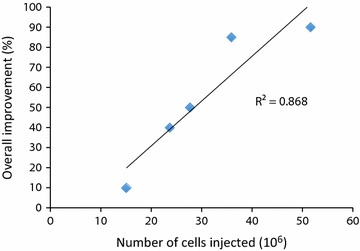
Total number of cells injected versus the overall improvement. Scatter plot displaying the overall improvement reported by patients under the quality of life questionnaires (values from Table 3) against the total number of cells injected into these patients (values from Table 1). The trend line shows positive correlation between the overall improvement and the total number of hypoxic cultured MSCs injected

Below are individual case reports on the five patients included in the study. Summarized details of the patients and treatments are provided in Table 1. Overall safety evaluations performed are depicted in Table 2. Summarized data from the quality of life questionnaire are provided in Table 3. Patient MRIs pre- and post-intra-discal injection, are presented in Fig. 1 and a scatter plot showing overall improvement versus number of hypoxic cultured MSCs injected, is presented in Fig. 2.

**Table 3:** Summary of the quality of life questionnaire data

Patient	1	2	3	4	5
Overall improvement (%)	50	90	40	10	80–90
Strength	Improved	Improved	Improved	Improved	Improved
Mobility	Improved	Improved	Improved	No change	Improved

### Patient 1

The patient is a 53-year-old female diagnosed with left thoracic outlet syndrome post trauma leading to chronic cervical and left arm pain. Patient also presented chronic lower back pain radiating into her leg on the left side extending down to the level of the knee. In March 2008, a pre-stem cell treatment baseline MRI of the lumbar region revealed that patient presented multilevel lumbar degenerative disc diseases at L4–L5 and L5–S1with a posterior disc height of 10 mm and a posterior disc protrusion of 2 mm at the latter (Fig. 1a left). After being explained the experimental nature of the proposed procedure, informed consent was obtained. In November 2009, this patient received an intra-discal injection of 27.68 million autologous, hypoxic cultured bone marrow-derived MSCs. Cells were injected into the posterior annulus fibrosus of the L5–S1 intervertebral disc. No complications occurred during the procedure and no adverse events were reported directly following the procedure. In May 2014, the patient returned for a follow-up examination consisting of a physical examination, completion of a quality of life questionnaire, and a lower back MRI. The lower back MRI post MSC transplantation, showed no neoplasms or abnormalities surrounding the treated region (Fig. 1b right). MRI measurements showed a mild progression of the disc degeneration with a posterior disc height of 8 mm and a 25 % increase in the posterior protrusion from 2 to 2.5 mm (Fig. 1a right). As part of the quality of life questionnaire, the patient self-reported an overall improvement of 50 % post stem cell transplant as well as an increase in strength and range of motion (Table 3). No adverse events were reported due to the stem cell procedure based on the quality of life questionnaire and the physical examination.

### Patient 2

The patient is a 25-year-old female with debilitating lower back pain with intermittent radiations down to her right leg. A pre-treatment MRI from July 2008 showed a significant reduction in disc height (8.5 mm), disc signal at L5–S1with a posterior disc protrusion of 4.3 mm (Fig. 1b left). Patient was explained the experimental nature of the procedure and signed an informed consent. This patient received a total of 51.6 million autologous, hypoxic cultured bone marrow-derived MSCs divided in two injections per patient request. She was transplanted with 14.3 million MSCs on July 2008 and with 37.3 million MSCs on June 2009. Cells were delivered into the right posterior annulus fibrosus and at the annular/nuclear interface of the L5–S1intervertebral disc. No complications occurred during the procedure and no adverse events were reported directly following the procedure. In April 2014 the patient returned for a follow-up examination consisting of a physical examination, completion of a quality of life questionnaire, and a lower back MRI. The lower back MRI post MSC transplantation showed no neoplasms or abnormalities surrounding the treated region. MRI measurements showed maintenance of disc height (8.8 mm) and a 20 % reduction of the posterior protrusion from 4.3 to 3.4 mm as compared to the pre- stem cell treatment MRI (Fig. 1b right). Based on the quality of life questionnaire, the patient self-reported an overall improvement of 90 % post stem cell transplant and an increase in strength and range of motion (Table 3). No adverse events were reported due to the stem cell procedure based on the quality of life questionnaire and the physical examination.

### Patient 3

The patient is a 40-year-old male with low back pain radiating into his right leg for 18 months prior to MSC injection. A baseline pre-stem cell treatment MRI imaging was performed on March 2010 and showed single L5–S1 level disc degeneration with a posterior disc height of 6.6 mm and a posterior disc protrusion of 3.5 mm (Fig. 1c left). After being explained the experimental nature of the proposed procedure, informed consent was obtained. On May 2010, this patient received an intra-discal injection of 23.7 million autologous, hypoxic cultured bone marrow-derived MSCs. Cells were injected into the L5–S1 intervertebral disc. No complications occurred during the procedure and no adverse events were reported directly following the procedure. The patient returned for a follow-up examination in July 2014 consisting of a physical examination, completion of a quality of life questionnaire, and a lumbar MRI. The lumbar MRI post-MSC transplantation showed no neoplasms or abnormalities surrounding the treated region. MRI measurement showed a mild decrease in disc height (5.6 mm) and a 43 % reduction of the posterior protrusion from 3.5 to 2.0 mm post stem cell treatment (Fig. 1c right). The patient self-reported an overall improvement of 40 % post stem cell transplant as well as an increase in strength and range of motion (Table 3). No adverse events were reported due to the stem cell procedure based on the quality of life questionnaire and the physical examination.

### Patient 4

The patient is a 43-year-old male with chronic lower lumbar and bilateral leg pain diagnosed with multilevel DDD in June 2008. In June 2009, a pre-stem cell treatment MRI, used as a baseline, showed a posterior disc height of 8.1 mm and the presence of a posterior protrusion of 4.1 mm with annular fissure and compression of the left S1 nerve root (Fig. 1d left). After being explained the experimental nature of the proposed procedure, informed consent was obtained. In September 2009, this patient received an intra-discal injection of 15.1 million autologous, hypoxic cultured bone marrow-derived MSCs. Cells were injected into the posterior annulus fibrosus of the L5–S1 intervertebral disc. No complications occurred during the procedure and no adverse events were reported directly following the procedure. In March 2014, the patient returned for a follow-up examination consisting of a physical examination, completion of a quality of life questionnaire, and a lower back MRI. MRI images post-MSC injection showed no neoplasms or abnormalities surrounding the treated region. MRI measurements at the L5–S1 level showed a mild progression of the posterior disc height loss (6.9 mm) and a 40 % reduction of the posterior protrusion from 4.1 to 2.5 mm post stem cell treatment (Fig. 1d right). The patient self-reported in the quality of life questionnaire an overall improvement of 10 % post stem cell transplant and an increase in strength while patient’s range of motion remained unchanged (Table 3). No adverse events were reported due to the stem cell procedure based on the quality of life questionnaire and the physical examination.

### Patient 5

The patient is a 41-year-old male with chronic lower back pain and bilateral radiculopathy more pronounced on the left side. In April 2009, a pretreatment MRI showed multilevel DDD from L3–L4 to L5–S1 levels, with disc protrusions at both L4–L5 and L5–S1 levels. At the L5–S1 level, the posterior disc height was measured at 5.8 mm and the protrusion at 6.5 mm (Fig. 1e left). After being explained the experimental nature of the proposed procedure, informed consent was obtained. In August 2009, this patient received an intra-discal injection of 35.9 million autologous, hypoxic cultured  bone marrow-derived MSCs. Cells were injected into the posterior annulus fibrosus of the L5–S1 intervertebral disc. No complications occurred during the procedure and no adverse events were reported directly following the procedure. The patient returned for a follow-up examination in July 2014 consisting of a physical examination, completion of a quality of life questionnaire, and a lumbar MRI. The lumbar MRI post MSC transplantation showed no neoplasms or abnormalities surrounding the treated region. MRI measurement showed maintenance of the posterior disc height (5.8 mm) and a 48 % reduction of the disc protrusion from 6.5 to 3.4 mm (Fig. 1e right). Based on the quality of life questionnaire, the patient self-reported an overall improvement of 80–90 % post stem cell transplant in addition to an increase in strength and range of motion (Table 3). No adverse events were reported due to the stem cell procedure based on the quality of life questionnaire and the physical examination.

## Discussion

There is a clear unmet medical need, prior to surgical intervention, for patients with chronic lower back pain associated with DDD. The standard of care including physiotherapy and corticosteroid epidural injections allows for pain management, at least initially, but does not frequently lead to prolonged pain relief and functional improvement. The degenerative cascade associated with DDD often leads over time to the aggravation of the symptoms at the affected disc level, but also to the deterioration of the adjacent intervertebral discs. After exhausting non-invasive options, patients face the prospect of surgery and, in particular, lumbar spinal fusion that does not guarantee complete pain alleviation and can present with significant complications and morbidity [30].

Regenerative medicine and stem cell therapies have gained tremendous interest in the past decades in multiple clinical applications and could represent a non-surgical biologic approach to improve pain and function in the millions of patients suffering from chronic low back pain associated with degenerative disc disease. Bone marrow-derived MSCs are of particular interest since they are easily accessible, they have been extensively studied, and they can be isolated following validated, standard operating procedures.

In the present study, we evaluated the safety and feasibility of an intra-discal injection of autologous, hypoxic cultured bone marrow-derived MSCs in five patients with chronic lower back pain. The five patients received between 15.1 and 51.6 million MSCs. These patients consented to a follow up exam 4–6 years post-MSC infusion consisting of a physical examination, completion of a quality of life questionnaire, and a lower back MRI. Data collected comprehensively showed that no adverse events, related to MSC infusion, occurred at the time of injection or up to 6 years post-injection, demonstrating long-term safety.

It is interesting to note that four out of five patients showed an improvement (reduction) of the protrusion size. Additionally all patients displayed maintenance or only mild worsening in disc height after long term follow up. Importantly, based on the quality of life questionnaires, the majority of patients reported an overall improvement, as well as an improvement in strength and mobility. Although this represents a small sample group, there appears to be a correlation between the total number of hypoxic cultured MSCs injected and the overall improvement reported by patients (Fig. 2).

Autologous, hypoxic cultured bone marrow-derived MSCs represent a potential attractive avenue for the regenerative treatment of patients with DDD. Preliminary safety data from the present study suggest that autologous, bone marrow-derived, hypoxic cultured MSCs are suitable for intra-discal injection of degenerated discs. Although these observations are encouraging, based on the small patient sample size and the uncontrolled nature of the study, a larger double-blind, controlled, randomized clinical study with significant number of patients and implementation of validated endpoint measurements such as the visual analog scale (VAS) pain scale and the functional rating index (FRI) test are next steps in order to demonstrate efficacy of this cell-based biologic.

## Conclusions

Intra-discal injection of autologous, hypoxic cultured bone marrow-derived MSCs demonstrated safety and feasibility in five patients diagnosed with degenerative disc disease. This data further support the clinical use of such MSCs for the treatment of lower back pain in larger clinical studies.

## Abbreviations

DDD: degenerative disc disease
MSC(s): mesenchymal stem cell(s)
MRI: magnetic resonance imaging
VAS: visual analog scale
FRI: functional rating index
QoL: quality of life
NP: nucleus pulposus
